# Study of Driving Fatigue Alleviation by Transcutaneous Acupoints Electrical Stimulations

**DOI:** 10.1155/2014/450249

**Published:** 2014-09-01

**Authors:** Fuwang Wang, Hong Wang

**Affiliations:** Department of Mechanical Engineering and Automation, Northeastern University, Shenyang, Liaoning 110819, China

## Abstract

Driving fatigue is more likely to bring serious safety trouble to traffic. Therefore, accurately and rapidly detecting driving fatigue state and alleviating fatigue are particularly important. In the present work, the electrical stimulation method stimulating the Láogóng point (劳宫PC8) of human body is proposed, which is used to alleviate the mental fatigue of drivers. The wavelet packet decomposition (WPD) is used to extract *θ*, *α*, and *β* subbands of drivers' electroencephalogram (EEG) signals. Performances of the two algorithms (*θ* + *α*)/(*α* + *β*) and *θ*/*β* are also assessed as possible indicators for fatigue detection. Finally, the differences between the drivers with electrical stimulation and normal driving are discussed. It is shown that stimulating the Láogóng point (劳宫PC8) using electrical stimulation method can alleviate driver fatigue effectively during longtime driving.

## 1. Introduction

Driving fatigue is a major problem in safety critical work situations as well as in public traffic [1, 2]. Statistics show that 15–20% of the total number of fatal traffic accidents is caused by driver fatigue [3–5]. Therefore, it is particularly important to detect driving fatigue and then alleviate this fatigue state efficiently. Researches have shown that the EEG, sensitive to neural activity [6–8], is considered to be an effective indicator for detecting driving fatigue [9].

Many methods can be used to alleviate driving fatigue, such as reducing the intensity of work, drinking caffeinated beverages, having a short rest, and taking special medicine. The electrical stimulation, which probably appeared in the 1960s, uses the appropriate low frequency electrical pulses to stimulate acupuncture points. In recent years, the electrical stimulation began to be applied in the research of counteracting the physiological fatigue [10]. Jian-ming Zhao has studied the stimulating points of human body to alleviate the physiological fatigue [11]. The transcutaneous acupoint electrical stimulation (TAES) has a great potential in counteracting mental fatigue. The transcutaneous acupoints which can alleviate mental fatigue include Neìguān point (内关PC6), Hégǔ point (合谷L14), Fēngchí point (风池GB20) [12], and Láogóng point (劳宫PC8) [13, 14]. In this paper, the method utilizing stimulating the Láogóng point (劳宫PC8) to relieve mental fatigue is used. In this method, the conductive cloth fixed on the car steering wheel is used as the stimulation electrode. Compared to the conventional electrical stimulation, the method does not need the patch electrodes, which makes it easy and convenient for using.

The main goal of the present study is a new method that may effectively alleviate the driving fatigue and is to be used commodiously. More specifically, the aim was to determine the effects: whether the transcutaneous acupoints electrical stimulations can alleviate driving fatigue during longtime driving in real driving condition. In our study, the electrical stimulation method stimulating the Láogóng point (劳宫PC8) of human body is proposed to alleviate the mental fatigue for drivers. The efficacy of electrical stimulation to inhibit human driving fatigue has been validated by comparing the significant differences between normal driving and TAES driving. In this paper, the *θ*, *α*, and *β* subbands of drivers' EEG signals are extracted using the WPD. Then, the relative power spectra (*θ* and *β*) and ratios (*θ*/*β* and *θ* + *α*/*α* + *β*) are calculated and analyzed for each driving stage. Finally, the differences of the parameters between the two groups' participants are analyzed.

## 2. Experiments

### 2.1. Subjects

A total of 22 healthy subjects (16 males and 6 females; aged 28 ± 1.6 (S.D.)) have been chosen for the experiment. All the subjects, free of medication during the experiment, were reported to have no sleep-related disorders or history of neurological diseases and asked to refrain from consuming any type of stimulus such as alcohol, tea, or coffee during the experiment.

### 2.2. Procedure

The Neuroscan, as the widespread use of EEG acquisition device, is very convenient to be used in a driving condition. And its electrodes (Ag/AgCl) are attached to the scalp according to the international 10–20 system (30 channels = FP1, FP2, F7, F3, FZ, F4, F8, FT7, FC3, FCZ, FC4, FT8, T3, C3, CZ,C4, T4, TP7, CP3, CPZ, CP4, TP8, T5, P3, PZ, P4, T6, O1, OZ, and O2). In the experiment, the Neuroscan device is chosen for EEG data recording. The EEG data recording for each stage lasts 3 minutes. Nine sets of data are collected for every participant. Recordings are performed with the ears mastoids (right and left) used as the common reference. In the experiment, it should be ensured that all leads are in a normal connection state during recording data for each stage. In addition, the electrical stimulation must be suspended during EEG signals recording.

**Figure 1:**
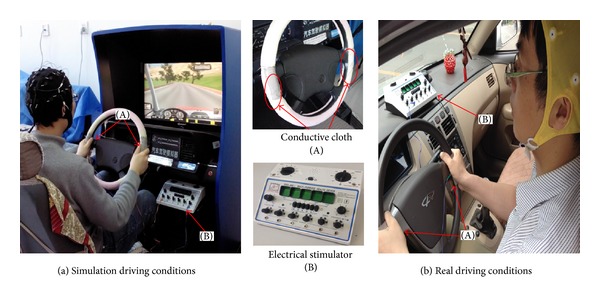
Experimental setup.

The multipurpose health device (KWD-808I), chosen as the electrical stimulator in the experiment, is used to stimulate transcutaneous acupoints. The stimulation current value with the range 1~3 mA is chosen by the participants on the basis of feeling comfortable, and the electrical stimulation pulse frequency is identified as 1 Hz. During the experiment, it must be ensured that the stimulation is below the levels that cause neural damage in cortical stimulation in animal and human studies.  Figure 1 shows the experimental setup.

The experiment was performed in simulation driving condition and real driving condition, respectively. The simulated experiment, performed in vehicle driving simulator, had chosen highway as experimental environment in a busy traffic situation on a sunny day. The experiment in real driving condition was performed on highway. In addition, the driving speed was selected in the range 80 km–90 km. The participants were divided into two groups. One group began the electrical stimulation after driving for an hour. The other one chose normal driving for the entire drive. Each group consists of eight males and three females. All subjects continuously drive for two hours (2:00 p.m.–4:00 p.m.). One hour of sleep (0:30 p.m.–1:30 p.m.) was arranged for all subjects to avoid the influence on fatigue due to the lack of sleep.

They were given one hour (0:30 p.m.–1:30 p.m.) for sleeping in the midday time to reduce the fatigue influence by sleeping. The process of data acquisition is divided into nine stages (stage 1—2:00 p.m., stage 2—2:15 p.m., stage 3—2:30 p.m., stage 4—2:45 p.m., stage 5—3:00 p.m., stage 6—3:15 p.m., stage7—3:30 p.m., stage 8—3:45 p.m., and stage 9—4:00 p.m.).

## 3. Methodology of Analysis

The process involves decomposing EEG signals into different bands using the wavelet pocket decomposition (WPD), calculation, and analysis of the relative power spectra (*θ* and *β*) and ratios (*θ*/*β* and *θ* + *α*/*α* + *β*) for driving process. The methods are explained in detail in the following sections.

### 3.1. Preprocessing and Artifact Removal Using WPD

The EEG recordings of drivers are easily influenced by noises. The noises mainly contain numerous low frequency and high frequency noises known as artifacts, such as the noises produced by the human body movement and the biological electrical signals. They should be filtered for the useful frequency band. In this paper, the subbands *θ*, *α*, and *β* were extracted from the raw EEG using the WPD method.

The WD can be represented as a continuous time wavelet decomposition sampled at different frequencies at every level. The wavelet decomposition functions at level *m* and time location *t*_*m*_ can be expressed as

(1) $$\begin{array}{c} d_{m}\left(t_{m}\right)=x\left(t\right)\psi_{m}\left(\frac{t-t_{m}}{2^{m}}\right), \end{array}$$

where Ψ_*m*_ is the decomposition filter at frequency level *m*. The effect of the decomposition filter is scaled by the factor 2*m* at stage *m*, but otherwise the shape is the same at all scales [15, 16]. The WD only partitions the frequency axis finely toward low frequency. However, the WPD is a generalized version, which also decomposes the high frequency bands which are kept intact in the WD [17]. The frequency resolution of the WD will descend with the increasing of the signal frequency. However, the WPD can overcome the defect and provide more precise analysis to signals. It can also select the appropriate subband according to the signal characteristics to match with signal spectrum. Therefore, the WPD can reflect the essential characteristics of signals and improve the time-frequency resolution. A WPD is represented as a function [18]:

(2) $$\begin{array}{c} \psi_{j+k}^{i}\left(t\right)=2^{-j/2}\psi^{j}\left(2^{-j}t-k\right), \end{array}$$

where *n* is the level of decomposition, *i* is the modulation parameter, *j* is the dilation parameter, *k* is the translation parameter, and *i* = 1,2,…, *j*_*n*_.

**Figure 2:**
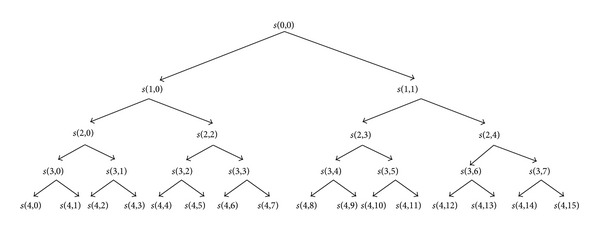
Wavelet packet decomposition tree.

The WPD, which decomposes both the low and the high frequency bands of signals, leads to a complete wavelet packet tree which is shown in Figure 2.

In this study, 4-layer decomposition is made using the WPD to extract *θ*(4~8 Hz), *α*(8~12 Hz), and *β*(12~32 Hz) rhythms. The *θ* wave (4~8 Hz) reconstructed based on subband *s*(4,1), the *α* wave (8~12 Hz) reconstructed based on subband *s*(4,2), and *β* wave (12~30 Hz) reconstructed based on subbands *s*(4,3), *s*(4,4), *s*(4,5), *s*(4,6), and *s*(4,7) are extracted from EEG.

### 3.2. Indicators for Detecting Driving Fatigue

The experimental process was segmented into 9 consecutive equal time sections. The data from the 9 stages were used to analyze the differences between normal driving and TAES. This analysis was performed for the theta and beta activities for the average of entire brain regions. Simultaneously, the algorithms *θ*/*β* and *θ* + *α*/*α* + *β* based on the relative power spectrum were separately analyzed for driving fatigue.

#### 3.2.1. The Relative Power Spectrum

For EEG analysis, *α* and *β* are deemed to be fast wave activities, whereas *δ* and *θ* are the slow wave activities. Studies have shown that the theta activity will increase during longtime monotony driving [19], and the beta activity will decrease during drowsiness [20]. In this study, the *θ* and *β* subbands are used to analyze the differences of driving fatigue between normal driving and TAES. For comparing conveniently, the data of the relative power spectrum are normalized by

(3) $$\begin{array}{c} s\left(i\right)=\frac{s\left(i\right)-\min\left(s\right)}{\max\left(s\right)-\min\left(s\right)}. \end{array}$$

#### 3.2.2. The Relative Power Spectrum Ratios

In the present work, the algorithms being analyzed are the relative power spectrum ratios between slow wave and fast wave activities. The power spectrum ratios for EEG have different combinations, such as *θ*/*β*, *θ*/*α* + *β*, *θ* + *α*/*β*, *θ* + *α*/*α* + *β*, and *β*/*α*, which can show different characteristics of driving fatigue over time [20–22]. From what has been discussed above, the relative power spectrum ratios (*θ*/*β* and *θ* + *α*/*α* + *β*) are used to analyze driver fatigue.

## 4. Results

### 4.1. The Relative Power Spectrum

Many studies have shown that the EEG signals can reflect the neural activity of human body [23, 24]. Many methods can be used to extract the characteristics of the EEG signals [24, 25]. The relative power spectra of subbands of EEG are the commonly used parameters in the analysis of driving fatigue. In this study, the relative power spectra *θ* and *β* were chosen as the indicators for analyzing driving fatigue. In order to analyze conveniently, the values of the relative power spectra *θ* and *β* were normalized firstly.

#### 4.1.1. Variation Tendencies with the Simulation Driving Condition

**Figure 3:**
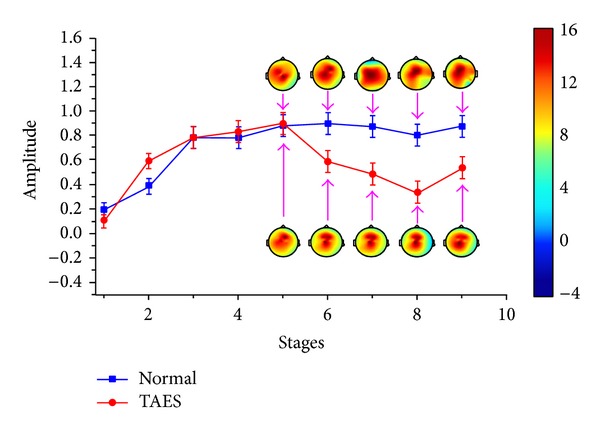
Relative power spectrum (mean ± S.D.) of *θ* subband for 9 stages of driving.

**Figure 4:**
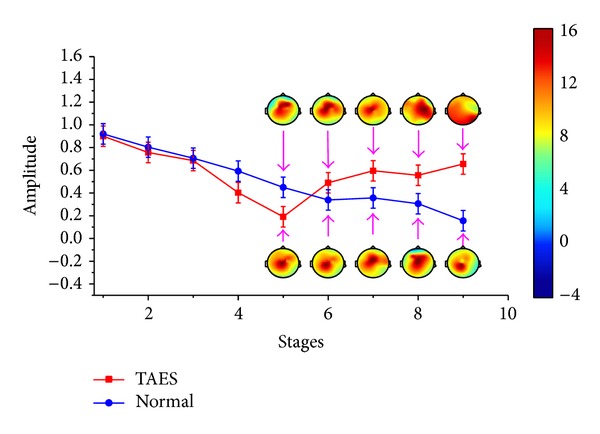
Relative power spectrum (mean ± S.D.) of *β* subband for 9 stages of driving.

In simulation driving condition, the characteristics of driving fatigue are significantly different between the drivers with TAES driving and normal driving (*P* < 0.05). These differences are shown as the variation tendencies of the relative power spectrum and the brain topography in *θ* and *β* subbands of EEG, which are shown in Figures 3 and 4.

Figure 3 shows that the relative power spectrum *θ*, for the participants with normal driving, presents general upward trend in the first three stages. From stage 4, the change tendency shows small fluctuations. For participants with TAES driving, the relative power spectrum *θ* presents a similar tendency like the participants with normal driving before stage 5 and a significant decline from stage 5 in which the electrical stimulator begins to work (*P* < 0.05).  Figure 4 also presents a significantly different change between the participants with TAES driving and normal driving. Although the relative power spectrum *β* to the two types of participants shows much alike change tendency in stages 1–5, the value from stage 5 to stage 9 presents a significant increase for participants with TAES driving and a continued decline for participants with normal driving.

Figures 3 and 4 also show the brain topography indicating theta and beta activities for one of the subjects. High activity is indicated by the red-shaded areas, whereas low activity is indicated by the blue-shaded areas. Figure 3 shows that the theta activity increased steadily for all brain regions for normal driving in driving stages 5–9, whereas the theta activity has a significant downward trend for all brain regions for TAES driving in driving stages 5–8.  Figure 4 presents that the beta activity decreased steadily for all brain regions for normal driving in driving stages 1–9, whereas the beta activity has a significant uptrend for all brain regions for TAES driving in driving stages 5–9.

#### 4.1.2. Variation Tendencies with the Real Driving Condition

**Figure 5:**
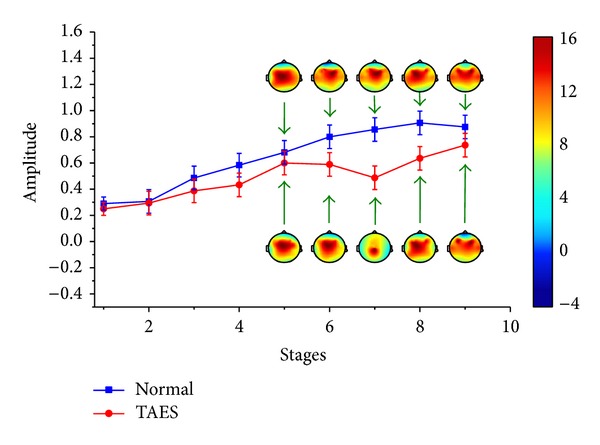
Relative power spectrum (mean ± S.D.) of *θ* subband for 9 stages of driving.

**Figure 6:**
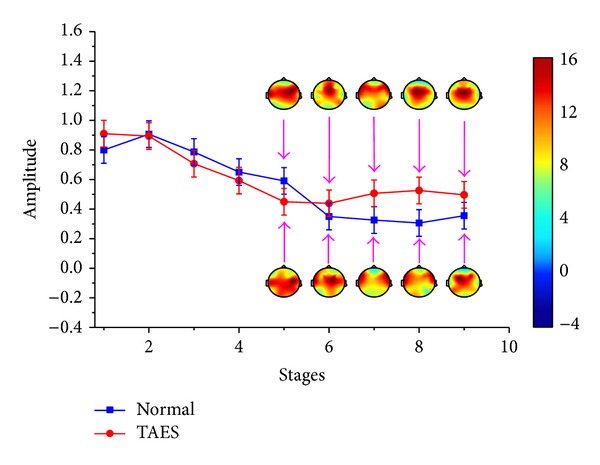
Relative power spectrum (mean ± S.D.) of *β* subband for 9 stages of driving.

Compared with simulation driving condition, the characteristics differences of driving fatigue are not so significant between the drivers with TAES driving and normal driving (*P* < 0.05). The variation tendencies are shown in Figures 5 and 6.

Figure 5 shows that the relative power spectrum *θ*, for the participants with normal driving, presents general upward trend in the process of the whole experiment. For participants with TAES driving, the relative power spectrum *θ* presents a similar tendency like the participants with normal driving before stage 5, a significant decline from stage 5 to stage 7, and a sharp rise from stage 7 to stage 9.  Figure 6 also presents the different changes between the two types of participants. A slow rising for participants with TAES driving and a continued decline for participants with normal driving can be easily observed in Figure 6 from stage 5 to stage 9.

Figures 5 and 6 also show the brain topography indicating theta and beta activities for the subjects. Figure 5 shows that the theta activity increased steadily for all brain regions for normal driving in driving stages 5–9, whereas the theta activity has a significant downward trend for all brain regions for TAES driving in driving stages 5–7 (*P* < 0.05) and a sharp increase in driving stages 7–9.  Figure 6 presents that the beta activity decreased steadily for all brain regions for normal driving in driving stages 1–9, whereas the beta activity has a slow uptrend for all brain regions for TAES driving in driving stages 5–9 (*P* < 0.05).

### 4.2. The Relative Power Spectrum Ratio

The relative power spectrum ratios *θ*/*β* and (*θ* + *α*)/(*β* + *α*) can reflect the changes of human brain activity, which are chosen as the indicators for analyzing driving fatigue.

#### 4.2.1. Variation Tendencies with the Simulation Driving Condition

**Figure 7:**
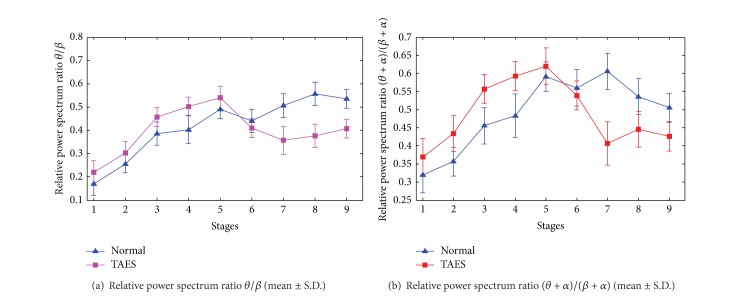
Ratios *θ*/*β* and (*θ* + *α*)/(*β* + *α*) of the relative power spectrum for 9 stages of driving.

In simulation driving condition, the relative power spectrum ratios are significantly different between the drivers with TAES driving and normal driving (*P* < 0.05). These differences of driving fatigue are shown in Figure 7.

Figure 7 shows that the ratios *θ*/*β* and (*θ* + *α*)/(*β* + *α*) of the relative power spectrum present significantly different changes between the participants with TAES driving and normal driving in stages 5–9 during the electrical stimulator working time. For participants with TAES driving, the relative power spectrum ratios *θ*/*β* and (*θ* + *α*)/(*β* + *α*) present a significant decline from stage 5 to stage 7 and a slow rise from stage 7 to stage 9, whereas, for the participants without TEAS, the ratios *θ*/*β* and (*θ* + *α*)/(*β* + *α*) present steady upward trend in all stages.

#### 4.2.2. Variation Tendencies with the Real Driving Condition

**Figure 8:**
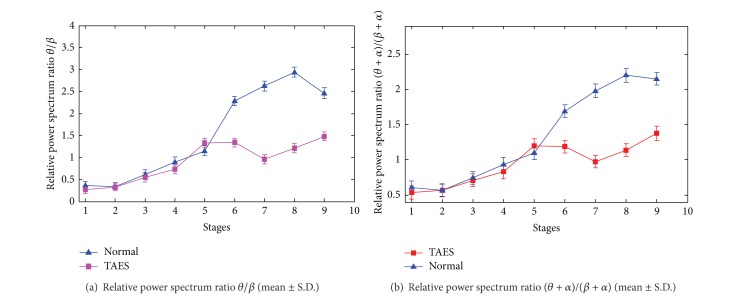
Ratios *θ*/*β* and (*θ* + *α*)/(*β* + *α*) of the relative power spectrum for 9 stages of driving.

Compared with simulation driving condition, the differences of variation tendencies of the relative power spectrum ratios between the drivers with TAES driving and normal driving are more significant in real driving condition (*P* < 0.05). These characteristics differences of driving fatigue are shown in Figure 8.

In real driving condition, Figure 8 shows that the ratios *θ*/*β* and (*θ* + *α*)/(*β* + *α*) of the relative power spectrum present significantly different changes between the participants with TAES driving and normal driving in stages 5–9 during the electrical stimulator working time. For participants with TAES driving, the relative power spectrum ratios *θ*/*β* and (*θ* + *α*)/(*β* + *α*) present a significant decline from stage 5 to stage 7 and a slow rise from stage 7 to stage 9, whereas, for the participants without TEAS, the ratios *θ*/*β* and (*θ* + *α*)/(*β* + *α*) present steady upward trend in all stages.

## 5. Discussions

Driver fatigue is more likely to bring serious safety trouble to traffic. Following increased fatigue, the unresponsive behaviors, decreased vigilance, and errors in judgment adversely affect safe driving. Therefore, relieving driving fatigue is particularly important for reducing the accidents caused by driving fatigue. In this paper, the method utilizing stimulating the Láogóng point (劳宫PC8) to relieve mental fatigue is used. The implications of data analysis are explored in the following discussion.

### 5.1. Electrical Stimulation Acupuncture Point

The needle stimulation is very helpful for relieving fatigue of human body. Studies have shown that transcutaneous electrical nerve stimulation can reduce pain and fatigue [10]. Stimulating the Neìguān point (内关PC6) and Fēngchí point (风池GB20) can relieve metal fatigue [13]. During longtime driving, stimulating the Neìguān point (内关PC6) can relieve driver fatigue [26]. From the view of traditional Chinese medicine (TCM), stimulating the Láogóng point (劳宫PC8) is helpful for relieving mental fatigue [27]. Although these methods can effectively relieve fatigue, the operation requires the patch electrodes which are not convenient for application in real driving. In this study, the method utilizing stimulating the Láogóng point (劳宫PC8) is used to relieve driving fatigue during longtime driving. Láogóng point (劳宫PC8) in the palm of the hand, close to the steering wheel when drivers are driving, is suitable for placing electrodes. The results show that the effect of relieving driving fatigue using this method is remarkable during longtime monotonous driving. Therefore, it can be considered a convenient and feasible method for relieving driving fatigue.

### 5.2. Features Detection for Driving Fatigue

Generally, the frequency band of EEG signals, which is in the range of 0.5 Hz–30 Hz, is used to analyze driving fatigue. And this band includes four frequency subbands *δ*(0–4 Hz), *θ*(4–8 Hz), *α*(8–12 Hz), and *β*(12–30 Hz). The EEG features of driving fatigue in the frequency band 0.5 Hz–30 Hz (including *δ*, *θ*, *α*, and *β*) have significant changes during longtime driving.

There are significant differences between drivers with TAES driving and normal driving (shown in Figures 3–8). Studies have shown that the *θ* activity will increase and the *β* activity will decrease with the mental fatigue gradually deepening [19, 20]. In the present work, it shows that the *θ* activity increases steadily (shown in Figures 3 and 5) and the *β* activity decreases steadily (shown in Figures 4 and 6) for drivers without TAES during longtime monotonous driving. On the contrary, for the drivers with TAES driving, there is a significant decrease in the *θ* activity (shown in Figures 3 and 5) and a steady increase in the *β* activity (shown in Figures 4 and 6) during longtime monotonous driving. It suggests that the fatigue caused by longtime monotonous driving can be eased after the subjects being given the electrical stimulation. Therefore, it can be concluded that stimulating Láogóng point (劳宫PC8) can relieve driver fatigue, which is helpful for improving safe driving for longtime driving. In addition, this method, which needs no stick electrodes, is convenient and practical to be used in actual driving situations, which provides a new method for relieving driving fatigue during longtime driving.

In simulation driving condition, the relative power spectrum *θ* shows a significant decline from stage 5 to stage 8 and a modest rise from stage 8 to stage 9 after the electrical stimulator working (shown in Figure 3). However, in real driving condition, the relative power spectrum *θ* presents a modest decline from stage 5 to stage 7 and a sharp rise from stage 7 to stage 9 (shown in Figure 5). The differences between the two driving conditions lie mainly in the length of valid time for relieving fatigue and the degree of easing fatigue. It can be seen obviously that the effect of relieving fatigue in simulation driving condition is more effective than in real driving condition. The cause of the difference of changes between the two types of conditions is due to the heavier workload in real driving condition compared with simulation condition. As we know, the drivers in real driving condition must deal with lots of information for safe driving, in which the body and mind are easier to become fatigued and are not easy to be relieved. In addition, for the relative power spectrum *β*, a significant increase for the drivers with TAES can be observed after electrical stimulator working in the two types of driving conditions (*P* < 0.05). That means that the activity of the mind and the brain cortex neurons of drivers becomes active; that is, the degree of the brain activity inhibited by the driving fatigue becomes lower.

## 6. Conclusions

In present work, the electrical stimulation method stimulating the Láogóng point (劳宫PC8) of human body is proposed, which is used to alleviate the mental fatigue of drivers. The *θ*, *α*, and *β* subbands are extracted from drivers' EEG signals. Then the differences between the drivers who are electrically stimulated and the drivers without electrical stimulation have been discussed. During longtime driving, the driving fatigue of the subjects without electrical stimulation increases gradually; on the contrary, the driving fatigue of the subjects can be alleviated when they were given electrical stimulation. The result shows that the electrical stimulation method stimulating the Láogóng point (劳宫PC8) of the human body can alleviate driving fatigue effectively. In addition, this method needs no stick electrodes, which is convenient and practical to be used in actual driving situations.
